# Theoretical Investigation of an Alcohol-Filled Tellurite Photonic Crystal Fiber Temperature Sensor Based on Four-Wave Mixing

**DOI:** 10.3390/s20041007

**Published:** 2020-02-13

**Authors:** Yue Sun, Xin Yan, Fang Wang, Xuenan Zhang, Shuguang Li, Takenobu Suzuki, Yasutake Ohishi, Tonglei Cheng

**Affiliations:** 1 State Key Laboratory of Synthetical Automation for Process Industries, College of Information Science and Engineering, Northeastern University, Shenyang 110819, China; 1800727@stu.neu.edu.cn (Y.S.); yanxin@ise.neu.edu.cn (X.Y.); wangfang@ise.neu.edu.cn (F.W.); zhangxuenan@ise.neu.edu.cn (X.Z.); lishuguang@ise.neu.edu.cn (S.L.); 2 Research Center for Advanced Photon Technology, Toyota Technological Institute, 2-12-1, Hisakata, Tempaku, Nagoya 468-8511, Japan; takenobu@toyota-ti.ac.jp (T.S.); ohishi@toyota-ti.ac.jp (Y.O.)

**Keywords:** temperature sensor, tellurite photonic crystal fiber, four-wave mixing, mid-infrared region

## Abstract

For this study, a temperature sensor utilizing a novel tellurite photonic crystal fiber (PCF) is designed. In order to improve the sensor sensitivity, alcohol is filled in the air holes of the tellurite PCF. Based on the degenerate four-wave mixing theory, temperature sensing in the mid-infrared region (MIR) can be achieved by detecting the wavelength shift of signal waves and idler waves during variations in temperature. Simulation results show that at a pump wavelength of 3550 nm, the temperature sensitivity of this proposed sensor can be as high as 0.70 nm/°C. To the best of our knowledge, this is the first study to propose temperature sensing in the MIR by drawing on four-wave mixing (FWM) in a non-silica PCF.

## 1. Introduction

Temperature sensors based on photonic crystal fibers (PCFs) have been a research hotspot in recent decades due to their small size and high temperature sensitivity [1]. In order to further improve the sensitivity, various methods have been adopted, such as based on fiber loop mirrors (FLMs) [2] and the modulation instability (MI) technique [3]. Materials other than the traditional silica dioxide have also been used to fabricate the PCFs [4], and temperature-sensitive materials, like oil [5], alcohol [6], liquid crystal [7], and silver nanowires [8,9], have been proposed for filling into the PCFs’ air holes.

Four-wave mixing (FWM), as an intermodulation phenomenon in nonlinear optics, is an alternative method to enhance the temperature-sensing sensitivity [10,11,12]. FWM originates from third-order nonlinear polarization of light and has been widely applied in fields, including wavelength division multiplexing [13,14], magnetic field sensing [15], strain sensing [16,17], and generation of a supercontinuum spectrum [18,19], to name a few. In optical fibers, at the occurrence of FWM, changes in temperature would induce the signal wave and idler wave to shift the wavelength, which can be utilized for temperature sensing.

Recently, tellurite glass has attracted extensive attention due to its unique features such as wide infrared transmission range, high nonlinear refractive index, high insulation constant, low melting temperature, low glass transition temperature (T_g_), and excellent third-order nonlinear optical properties [20]. Possessing a refractive index of ~2.0 [21], tellurite glass fibers could support light transmission in near-infrared (NIR) and mid-infrared (MIR) regions, which is impossible for traditional silicon material due to its large loss. The tellurite material provides a good platform for the generation of FWM and offers an opportunity for temperature sensing in the MIR.

In this paper, a temperature sensor utilizing a tellurite PCF is designed based on FWM. In order to achieve MIR temperature sensing, we optimize the fiber parameters and design the fiber structure to be solid with the exception of three adjacent holes filled with alcohol in Mode Solution software. According to the amount of degenerated FWM, temperature sensing can be realized by detecting the wavelength drift of signal wave and idler wave at the change of temperature. Through the use of MATLAB software programming, the sensitivity of this temperature sensor can reach 0.70 nm/°C at a pump wavelength of 3500 nm. Being simple in structure and high in sensitivity, this sensor could be used for light induction through human body MIR radiation detection.

## 2. Materials and Methods

### 2.1. Structure Design of the PCF

**Figure 1:**
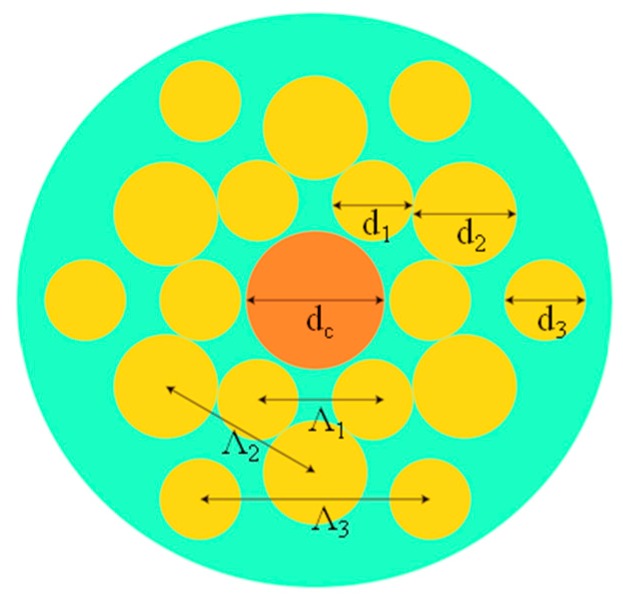
Structure of tellurite photonic crystal fiber (PCF). d_c_ is the core diameter, d_1_, d_2_, and d_3_ are the diameter of the rods in the first layer, the second layer, and the third layer, respectively. Λ_1_, Λ_2_, and Λ_3_ are respectively the size of rods spacing in the first layer, the second layer, and the third layer.

The tellurite PCF used for the proposed sensor consists of two kinds of tellurite materials: TeO_2_-ZnO-Na_2_O-P_2_O_5_ (TZNP) for the cladding and TeO_2_-LiO_2_-WO_3_-MoO_3_-Nb_2_O_5_ (TLWMN) for the fiber core and rods [22]. The component proportion of TLWMN for the rods is slightly different from that for the core, leading to a refractive index difference of 0.025. The core diameter is indicated by d_c_, and d_1_, d_2_, and d_3_ are the diameters of the first layer rods, the second layer rods and the third layer rods, respectively. Λ_1_, Λ_2_, and Λ_3_ are the sizes of rod spacing in the first layer, the second layer, and the third layer in Figure 1.

To realize temperature sensing in the MIR, we need to generate FWM in the MIR, which requires the tellurite PCF to possess a dispersion curve with zero dispersion wavelength (ZDW) and be as flat as possible in the MIR. For this purpose, the dispersion curve is simulated within the MIR range of 2500 to 4000 nm by respectively changing the core diameter (d_c_) and the rod diameters (d_1_, d_2_, and d_3_). Λ_1_ is fixed to be 2 μm, Λ_2_ 2√3 μm, and Λ_3_ 4 μm.

**Figure 2:**
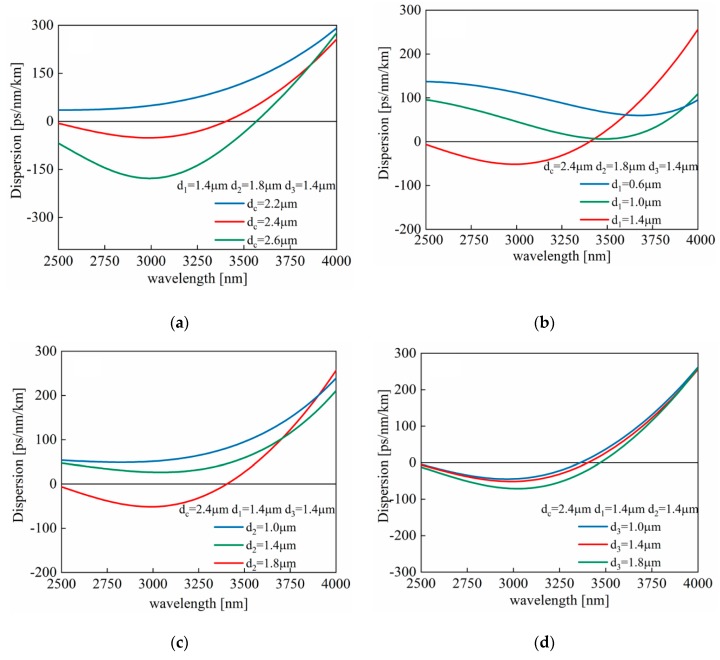
Dispersion curves of tellurite PCF for various values of (**a**) d_c_; (**b**) d_1_; (**c**) d_2_; (**d**) d_3_. Λ_1_, Λ_2_, Λ_3_ are 2, 2√3, and 4 μm respectively.

Figure 2a shows the calculated dispersion curves with variation of d_c_ (2, 2.2, and 2.4 μm). Other parameters are as follows: d_1_ = 1.4 μm, d_2_ = 1.8 μm, d_3_ = 1.4 μm. We can see that with the increase of d_c_, the ZDW appears in the wavelength range of 2500 to 4000 nm while the dispersion curves become less flat. As a result, d_c_ is 2.4 μm, which is the most desirable. Similarly, the dispersion curves with the variation of d_1_ (0.6, 1, and 1.4 μm) and d_2_ (1, 1.4, and 1.8 μm) are respectively shown by Figure 2b,c. Parameters d_1_ = 1.4 μm, d_2_ = 1.8 μm are selected.

Figure 2d illustrates the curve with the variation of d_3_ while d_c_ = 2.4 μm, d_1_ = 1.4 μm, d_2_ = 1.8 μm. It can be seen that the smaller the rod diameter, the flatter the dispersion curve. However, when d_3_ is too small, the dispersion curve brings out a few changes, which means that the PCF’s binding force to the light is extremely weak, leading to more light being transmitted in the cladding and loss value increasing. Therefore, there is a need to control the diameter of the third layer rods and ensure that PCF has sufficient restraint on light and a dispersion curve as relatively flat as possible. In order to achieve these two points, and taking advantage of comparing the dispersion and loss values obtained by changing the diameter of the third layer rods, d_3_ was chosen as 1.4 μm.

### 2.2. Structure Design of the Temperature sEnsor

Based on the fiber parameters of d_c_ = 2.4 μm, d_1_ = 1.4 μm, d_2_ = 1.8 μm. d_3_ = 1.4 μm, Λ_1_ = 2 μm, Λ_2_ = 2√3 μm, and Λ_3_ = 4 μm, the tellurite PCF was designed as a temperature sensor. However, the tellurite glass has a thermo-optic coefficient of minus six orders of magnitude, which is not beneficial for temperature sensing. To overcome this problem, it was proposed to replace certain rods with air holes filled with temperature-sensitive material: alcohol. The thermo-optic coefficient of alcohol is ξ = Δn/ΔT = −4 × 10^−4^/°C, which is two orders of magnitude higher than that of tellurite glass. By replacing the solid rod with alcohol-filled air holes, the quantitative change of the refractive index of tellurite glass with temperature variation can be ignored during the calculation of the effective refraction index. The refractive index of alcohol can be calculated as a function of temperature. The original refractive index of alcohol is shown by [23]. On the basis of formula

n_new_ = n_original_ + Δn = n_original_ + ξ × ΔT, (1)

Relevant fiber parameters such as effective refractive index, nonlinear coefficient, and dispersion curve can be calculated using the finite element method.

**Figure 3:**
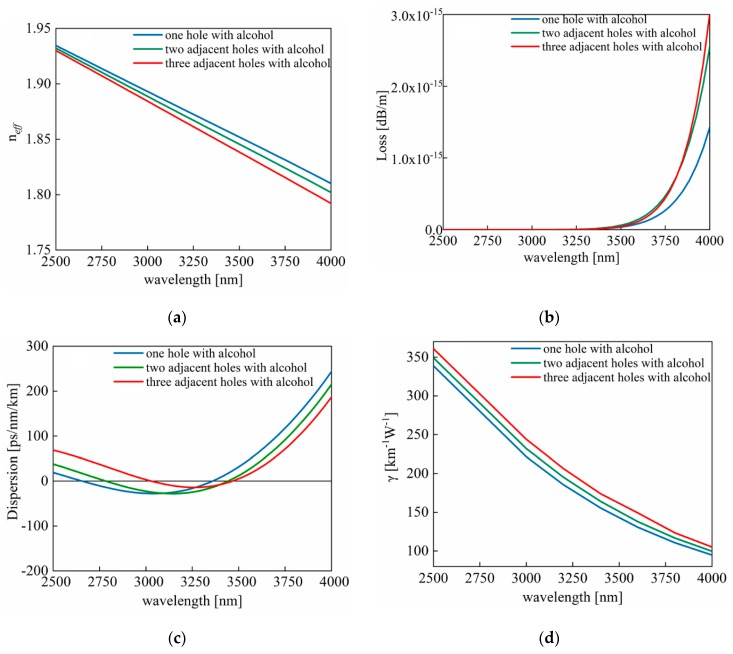
(**a**) Calculated effective index curves of one hole, two adjacent holes, and three adjacent holes with alcohol in the first layer, respectively. (**b**) Loss curves of three filling methods. (**c**) Dispersion curves of three filling means. (**d**) Nonlinear coefficient of three filling methods.

In the next step, one, two, and three glass rods in the first layer are respectively replaced by air holes filled with alcohol, and ΔT = 0 °C. Figure 3a,b, gives information about the effective refractive index and the loss curves of these three replacement cases at wavelengths ranging from 2500 to 4000 nm. It is clear that in spite of increasing the number of alcohol-filled air holes, the loss presents no significant disparity. Figure 3c describes the calculated dispersion curves of the three replacement cases. It is to be noted that when adopting three alcohol-filled air holes, the dispersion curve is much flatter, and it is not closer to zero than the other two filling methods at 2500 to 3000 nm but closer to zero at 3000 to 4000 nm. In order to achieve FWM more easily, we chose filling three holes and controlled the pump wavelength in the range from 3000 to 3600 nm, which is the flattest part of this dispersion curve. Additionally, there are normal dispersion region and abnormal dispersion region which can make contributions to the comparison of temperature sensing in the different dispersion regions. Figure 3d demonstrates the calculated nonlinear coefficients whose values have an overall increase with the growth of the replacement number. For the degenerate FWM, g = √(γP_0_)^2^ − (κ/2)^2^. When the phase-matching (PM) condition is satisfied, the theoretical maximum gain is γP_0_. As a result, the larger the nonlinear coefficient γ, the bigger the gain coefficient g. When replacing three adjacent rods in the first layer, the nonlinear coefficient is the largest, so is the gain at the satisfaction of PM condition. Therefore, by replacing three solid rods in the first layer with alcohol-filled air holes temperature sensing is accomplished.

As can be seen from the above, when the structural parameters of the tellurite PCF are d_c_ = 2.4 μm, d_1_ = 1.4 μm, d_2_ = 1.8 μm, d_3_ = 1.4 μm, Λ_1_ = 2 μm, Λ_2_ = 2√3 μm, and Λ_3_ = 4 μm, a flattened dispersion curve with one ZDW in the MIR can be obtained within the wavelength range of 2500 to 4000 nm. Owing to the insensitivity of tellurite glass to temperature, we take out the rods and replace them with air holes filled with alcohol. Through comparing three different filling methods, it is clear that the curve obtained from three adjacent glass rods replaced with air holes filled with alcohol in the first layer is relatively flat. In the following work, the pump wavelength from 3000 to 3600 nm was selected. With three holes filled, dispersion in this wavelength range is closest to zero over the entire range. It is beneficial to satisfy the PM condition and realize FWM in the MIR.

## 3. Results

On the basis of FWM theory, when the PM condition is satisfied, the change of temperature (ΔT) can induce a shift in the signal wavelength, which can be utilized as a means to realize temperature sensing. PM condition is given by

Κ = Δk + 2γP_0_ = 0 (2)

where κ and ∆k are, respectively, the nonlinear and linear phase mismatch, γ is the nonlinear coefficient, and P_0_ is the sum of two pump powers.

**Figure 4:**
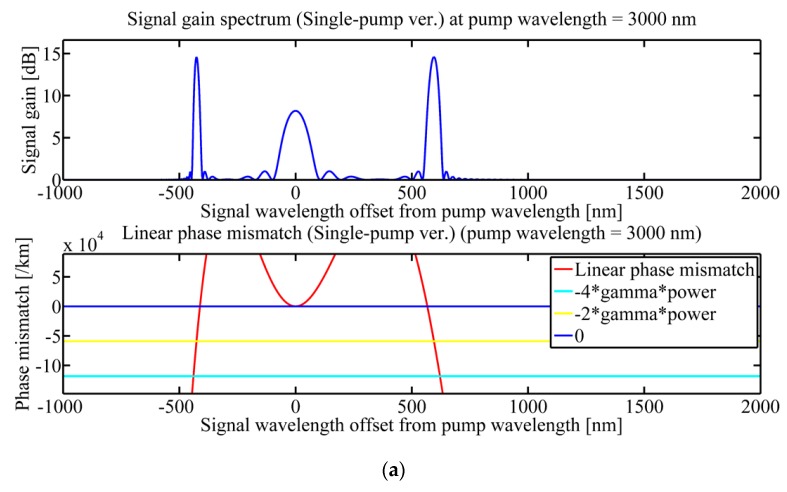

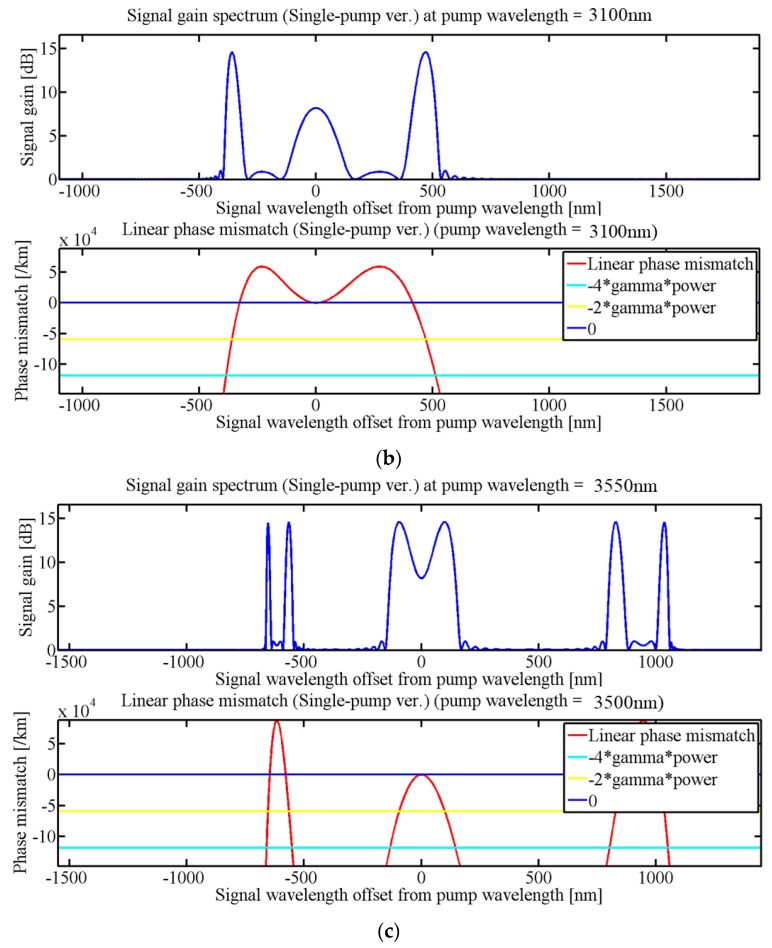
Optical signal gain intensity and phase matching diagram at different pump wavelengths (**a**) 3000 nm; (**b**) 3100 nm; (**c**) 3500 nm.

Different pumping wavelengths were selected within the range of 3000 to 3600 nm to detect the sensor’s temperature sensitivity in the MIR, which were 3000, 3100, and 3550 nm. The pump power (P_0_) is 100 W and the fiber length is 8 cm. Firstly, ΔT = 0 °C, and the optical signal gain intensity and PM diagram obtained at these three pumping wavelengths are given in Figure 4.

In each group of figures, the intersections of the red line and the yellow line meet the PM condition. These intersection points correspond to the maximum peak value on the left and right side of the blue curve in each group of images, which are idler gain peak and signal gain peak, respectively. At 3550 nm, two PM conditions are satisfied, which induce two pairs of signal waves and idler waves. We call the signal and idler wave near the pumping wavelength as the first-order signal/idler wave while those far away as the second-order signal/idler wave. The generation of two pairs of signal and idler waves could produce more nonlinear effects.

**Figure 5:**
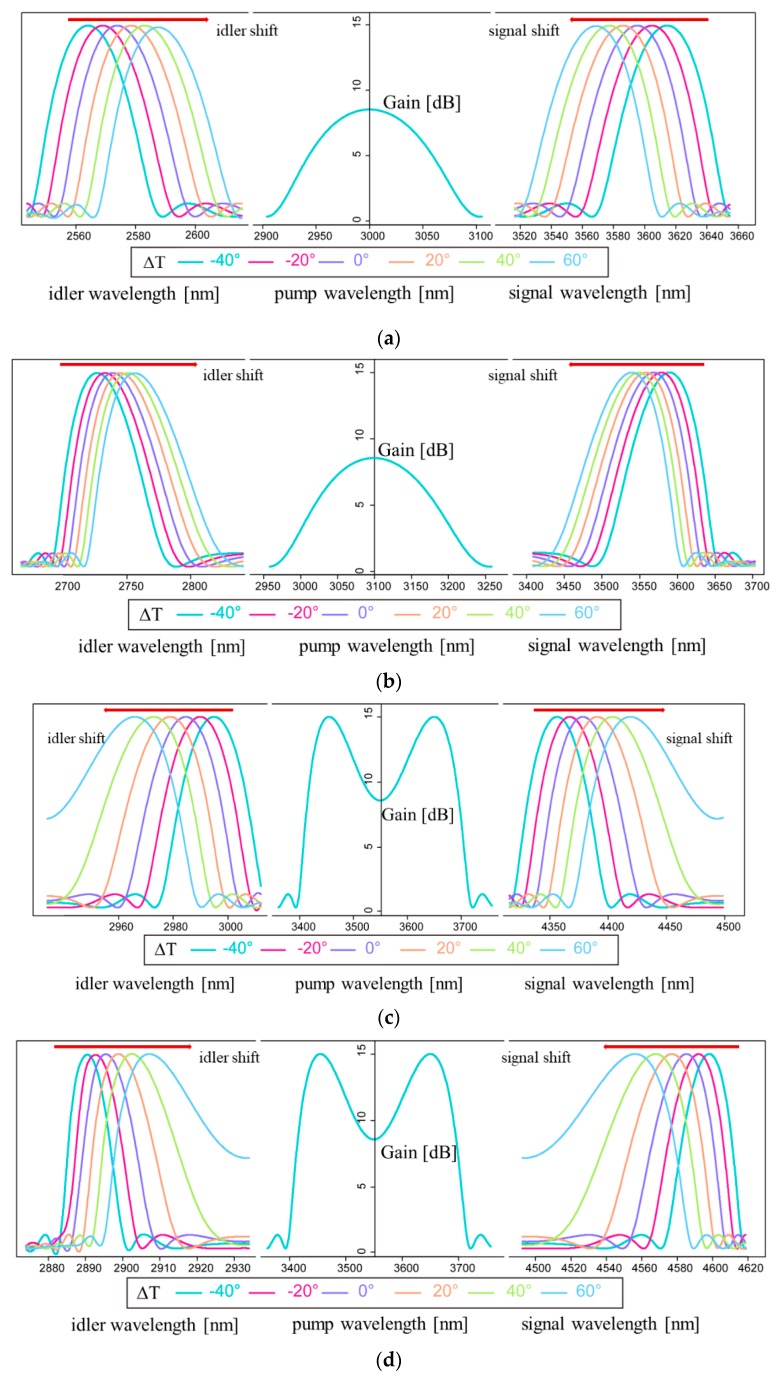
Theoretical spectrum of idler and signal wavelength shift with temperature change from 40 to 60 °C at different pump wavelengths. (**a**) 3000 nm; (**b**) 3100 nm; (**c**) 3550 nm (wavelength shift of the first signal wave and idler wave); (**d**) 3550 nm (wavelength shift of the second signal wave and idler wave).

Furthermore, the four graphs in Figure 5 are the curves of signal waves and idler waves moving as temperature changes. Figure 5a illustrates the wavelengths shift when the pump wavelength is 3000 nm. At this time, the dispersion value is in the abnormal dispersion region. The calculated sensitivities of the signal wave and the idler wave are 0.46 and 0.23 nm/°C, respectively. Similarly, Figure 5b shows that the pump wavelength is 3100 nm, and the dispersion value is in the normal dispersion region. As can be seen from the figure, the temperature sensitivity of the signal wave is 0.50 nm/°C, while the sensitivity of the idler wave is 0.30 nm/°C. As shown in Figure 5c,d, the dispersion value is in the anomalous dispersion region. After calculation, the temperature sensitivity of the first-order signal wave is 0.70 nm/°C, and that of the idler wave is 0.29 nm/°C. The temperature sensitivities of the second-order signal wave and idler wave are 0.41 and 0.17 nm/°C, respectively.

**Figure 6:**
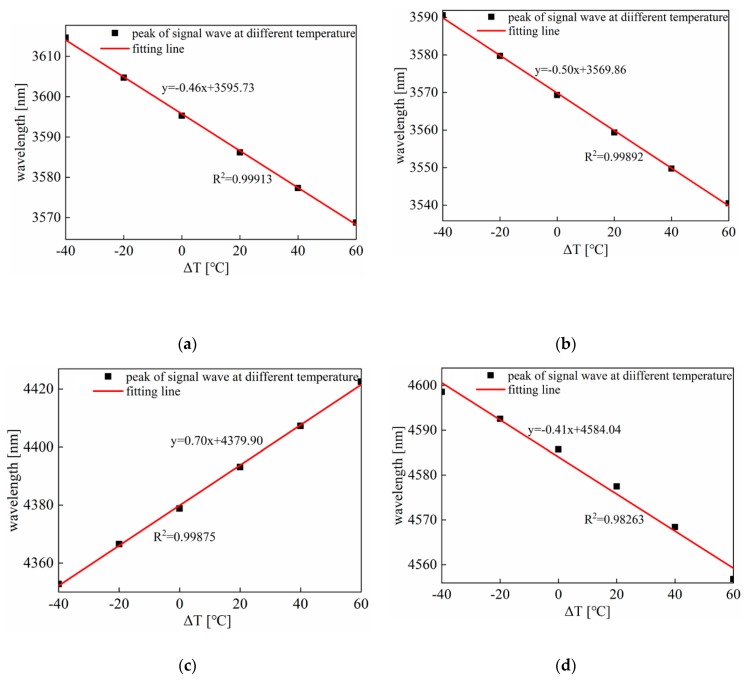
The peak movement of the signal wave varies with temperature at different pump wavelengths. (**a**) 3000 nm; (**b**) 3100 nm; (**c**) 3550 nm (the first-order signal waves generate); (**d**) 3550 nm (the second-order signal waves generate).

Figure 6 describes the function of signal wavelength with the temperature at the pump wavelengths of 3000, 3100, and 3550 nm. The obtained signal crests have a good linear relationship with the temperature (ΔT), so the signal wavelengths which shift with the changes in temperature wavelengths can be utilized in temperature sensing. From the above theoretical analysis, it can be concluded that when pump wavelength is 3550 nm, the temperature sensitivity of the proposed sensor is the highest, which can reach 0.70 nm/°C at ΔT changing from −40 to 60 °C.

## 4. Discussion

Table 1 compares the performance of our proposed temperature sensor with those reported previously. It is clear that the FWM-based temperature sensor has a sensitivity much higher than that of [4,14,24,25], and is only lower than that of [26]. However, the detection range of our work is −40~60 °C, which is five times that of [26] (20~40 °C). Additionally, the fiber proposed in [26] is a gold-coated PCF, and the thickness of its gold film cannot be controlled accurately in real practice, which may greatly influence its temperature sensitivity. This study clearly shows the efficiency of FWM in a tellurite PCF for temperature sensing, which not only obtains higher sensitivity but also realizes the temperature sensing in the MIR.

## 5. Conclusions

In this paper, by carefully designing the fiber parameters of a tellurite PCF and further improving the fiber structure, a temperature sensor with high sensitivity in the MIR has been designed. It has a solid structure except for three adjacent holes filled with alcohol to avoid the difficulty of selective filling in the experiment. Unlike traditional fiber-optic temperature sensors, we draw on the FWM to realize temperature sensing. When pumped at 3550 nm in the MIR, this sensor achieves a sensing sensitivity as high as 0.70 nm/°C. It can be applied in fields such as fingerprint unlocking and the body radiation photosensitive system. Due to the limitation of experimental conditions, this work only provides the theoretical simulation and analysis, which hopefully lays a good foundation for the development of MIR optical sensing devices in the future.

## Figures and Tables

**Table 1:** Comparison with predecessors.

Type of Sensor	Sensitivity	Reference	Detection Range
A solid core photonic crystal fiber filled with a layer based on FWM	0.61 nm/°C	[4]	30 °C–170 °C
A photonic crystal fiber based on FWM with oil-filled	0.207 nm/°C	[14]	22 °C–72 °C
Fabry-Perot interferometer	−0.029 nm/°C	[24]	0 °C–50 °C
D-shaped gold coated PCF	12.31 nm/°C	[26]	20 °C–40 °C
Soft glass fiber Bragg grating sensor	0.1757 pm/°C	[25]	0 °C–200 °C
Our tellurite PCF based on FWM	0.70 nm/°C	This work	−40 °C–60 °C(ΔT)
